# Expression profiles and prognostic significance of AFTPH in different tumors

**DOI:** 10.1002/2211-5463.13003

**Published:** 2020-10-28

**Authors:** Tengjiao Zhu, Yingtong Chen, Zhongjun Liu, Yuxin Leng, Yun Tian

**Affiliations:** ^1^ Third Hospital of Peking University Beijing China

**Keywords:** AFTPH, bioinformatics, cancer, prognosis

## Abstract

Aftiphilin (AFTPH) plays an important role in regulating intracellular trafficking, exocytosis, and the pro‐inflammatory response. However, the potential prognostic role of AFTPH in cancers remains unclear. Here, we examined the expression profiles and prognostic significance of AFTPH in breast invasive carcinoma (BRCA), diffuse large B‐cell lymphoma (DLBC), lung squamous cell carcinoma (LUSC), and pancreatic adenocarcinoma (PADD) using the GEPIA and UALCAN databases. AFTPH expression was observed to be higher in cancer tissues than in normal tissues, but expression did not differ significantly between tumor stages for the four cancer types. AFTPH expression in cancer cell lines was investigated using the CCLE database; AFTPH was found to be highly expressed in four cancer cell lines. The relationship between AFTPH expression and patient prognosis was analyzed using GEPIA, LinkedOmics, and Kaplan–Meier plotter databases. Low expression of AFTPH was associated with improved prognosis for BRCA, DLBC, LUSC, and PAAD. Genetic alterations of AFTPH in cancers were explored using the cBioPortal website, revealing that gene copy number gains and amplification are common in BRCA, DLBC, LUSC, and PAAD. Related genes and markers associated with AFTPH were discovered using the LinkedOmics database. Furthermore, transfection of cells with AFTPH siRNA demonstrated that AFTPH exerts positive effects on cell proliferation in BRCA, LUSC, and PAAD cells. In conclusion, AFTPH may be a potential therapeutic target and prognostic biomarker for BRCA, DLBC, LUSC, and/or PAAD.

## Graphical abstract

Aftiphilin (AFTPH) plays an important role in regulating intracellular trafficking, exocytosis, and pro‐inflammatory response. The potential role of AFTPH in cancers remains unclear. This study was the first to comprehensively analyze the expression profiles and prognostic role of AFTPH in solid tumors and hematological malignancies, as well as providing evidence for subsequent studies on the molecular mechanism governing this role. AFTPH was suggested to be a potential therapeutic target and prognostic biomarker for BRCA, DLBC, LUSC, and PAAD.

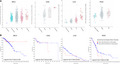

**Abbreviations**
BRCA: breast invasive carcinoma
CCLE: cancer cell line encyclopedia
DLBCL: diffuse large B‐cell lymphoma
GEPIA: gene expression profiling interactive analysis
HPA: human protein atlas
LUSC: lung squamous cell carcinoma
PADD: pancreatic adenocarcinoma

Cancer has become a global public health issue. It is estimated that there are more than 1.7 million newly diagnosed cases and 0.06 million cancer‐related deaths in 2019. The morbidity of lung squamous cell carcinoma (LUSC) ranks second among all malignant tumors, and LUSC is the major cause of death from cancer. Breast invasive carcinoma (BRCA) is the most common malignancy and the second leading cause of cancer‐related death in women. In addition, pancreatic adenocarcinoma (PADD) and diffuse large B‐cell lymphoma (DLBC) are frequent cancer types in both males and females [1]. Finding a popular cancer biomarker is a useful means of achieving early diagnosis and treatment of cancer, which may reduce mortality.

Aftiphilin (AFTPH) is one of the accessory proteins of the heterotetrameric adaptor complex 1 (AP‐1) in mammals. AFTPH can bind itself to the γ‐adaptin ear domain of AP‐1 via clathrin‐binding motifs, contributing to the trans‐Golgi network (TGN) and endosomal clathrin coats [2]. AFTPH has been verified as a component of the clathrin‐coated vesicle (CCV) machinery and is involved in the mechanism of clathrin‐mediated membrane budding in neurons [3, 4]. The interaction between AFTPH and AP‐1 plays an important role in the eye development of *Drosophila* [5]. Deletion of AFTPH and γ‐synergin has a negative effect on the secretory organelles in epithelial cells [6]. In recent studies, AFTPH was discovered to be a downstream target of miR‐133α, playing a role in neurotensin (NT)/NT receptor 1 (NTR1)‐mediated colonic inflammatory signaling and NTR1 trafficking in colonic epithelial cells [7, 8, 9].

To date, no research has focused on AFTPH expression in human cancers. Therefore, our study underscored the importance of the specific role of AFTPH in tumors, including BRCA, DLBC, LUSC, and PAAD, with the help of bioinformatic tools. The expression and alterations of AFTPH in cancers, the relationship between AFTPH and patients with different clinicopathological characteristics, the prognostic impact of AFTPH, the potential interaction of AFTPH with related genes, the immune infiltrating types, and the effect of AFTPH on cell proliferation were primarily discussed.

## Materials and methods

### GEPIA database analysis

The GEPIA database (Gene Expression Profiling Interactive Analysis, http://gepia.cancer‐pku.cn/index.html) is a public database for detecting gene expression profiles in tumor samples and normal samples. The database can be used to analyze RNA sequencing expression data of 9736 tumors and 8587 normal samples from TCGA and GTEx [10]. In this study, comparison of AFTPH expression in tumor tissues and normal tissues, as well as distinct tumor stages and determination of the relationship between AFTPH expression and patient survival, was performed via GEPIA.

### UALCAN database analysis

UALCAN database (http://ualcan.path.uab.edu/) is a data mining website, where the gene expression situation in tumors can be queried and prognostic information can be obtained [11]. In this study, UALCAN was used to explore AFTPH expression in BRCA and LUSC and corresponding adjacent tissues, as well as the effect of AFTPH expression level on BRCA patient survival.

### HPA database analysis

The HPA database (Human Protein Atlas, www.proteinatlas.org.) is the largest and most comprehensive database for evaluating protein distribution in human tissues and cells, consisting of the Tissue Atlas, the Pathology Atlas, and the Cell Atlas. Protein expression and localization in tissues and specific gene expression in cell lines can be determined through RNA sequencing and immunohistochemistry [12]. In this study, immunohistochemical staining of AFTPH in BRCA and LUSC was assessed by the HPA database.

### CCLE database analysis

The CCLE (Cancer Cell Line Encyclopedia, http://www.broadinstitute.org/ccle/home) is a public source that provides gene expression, chromosomal copy number, and massively parallel sequencing data of more than 1000 cancer cell lines [13]. In this study, CCLE was used to compare the expression levels of AFTPH in various cell lines.

### LinkedOmics database analysis

LinkedOmics database (http://www.linkedomics.org), containing three analysis modules of LinkFinder, LinkCompare, and LinkInterpreter, is a public website for obtaining multiomic data of primary tumors based on 32 cancer types from TCGA (The Cancer Genome Atlas) and 11 158 patients. Available information includes gene mutations, copy number alterations, DNA methylation, mRNA expression, and global proteomics data [14]. In this study, LinkedOmics was used to analyze the correlation between AFTPH and other genes.

### Kaplan–Meier plotter analysis

Kaplan–Meier plotter (www.kmplot.com) is an open source database that provides genomic profiles and survival conditions in 21 kinds of cancer [15]. In this study, the relationship between AFTPH expression level and clinical outcomes in PAAD patients was generated by Kaplan–Meier survival analysis. Hazard ratios (HRs) with 95% confidence intervals and log‐rank *P*‐values were also calculated and presented.

### cBioPortal analysis

The cBioPortal database (cBioPortal for Cancer Genomics, http://cbioportal.org) is an online database that converts complex cancer genomics data from TCGA into well‐understood genetic, epigenetic, and proteomic data, including somatic mutations, altered copy number, mRNA and miRNA expression, DNA methylation, and protein abundance data. The database can be used to explore genetic changes in tumor samples and compare the effects of these changes on patient survival [16]. In this study, AFTPH genetic changes in BRCA, DLBC, LUSC, and PAAD were revealed by cBioPortal. Survival curves were created to visualize the relationship between AFTPH gene changes and clinical prognosis.

### Cell lines and cell culture

The BRCA cell line MCF7, LUSC cell line A549, and PAAD cell line PANC1 (purchased from China Infrastructure of Cell Line Resources, Beijing, China) were selected for the following experiments. The cells were cultured in RPMI1640 medium (Gibco, Grand Island, NY, USA) supplemented with 10% fetal bovine serum (Gibco), 100 μ·mL^−1^ penicillin, and 100 μg·mL^−1^ streptomycin. The cells were maintained at 37°C in a humidified atmosphere containing 5% CO_2_.

### Transfection

To explore the loss of function of AFTPH, MCF7, A549, and PANC1, cells were transfected with AFTPH siRNA (synthesized by JiMa) using GP‐transfect‐Mate (purchased from JiMa) for 2 consecutive days. The sequences of AFTPH siRNA were as follows: sense 5' GCAGGCUACUGAAUCUCAUTT 3' and antisense 5' AUGAGAUUCAGUAGCCUGCTT 3'. The sequences of NC siRNA were as follows: sense 5' UUCUCCGAACGUGUCACGUTT 3' and antisense 5' ACGUGACACGUUCGGAGAATT 3'. The cells were harvested after 48 h of transfection for further experiments.

### RNA isolation and reverse transcription quantitative polymerase chain reaction

Whole RNA of control and AFTPH siRNA cells was extracted and reverse‐transcribed into cDNA using the FastKing First Strand cDNA Synthesis Kit (TIANGEN, Suzhou, China) according to the instructions. Then, real‐time quantitative PCR was performed in triplicate with β‐actin as an endogenous control. The Livak method was used to calculate the level of AFTPH mRNA. Specific primer sequences for AFTPH and β‐actin are indicated in Table 1.

**Table 1:** Primer sequences

Gene	Direction	Sequence (5’‐3’)
AFTPH	Forward	TTTGGAGACCAGCAGGCTACT
	Reverse	TTGGGGGTTCCTGGAGTATCA
β‐actin	Forward	GCGTGACATTAAGGAGAAG
	Reverse	GAAGGAAGGCTGGAAGAG

### Cell proliferation assay

After 2 consecutive days of transfection, 5 × 10^3^ cells were seeded into each well of a 96‐well plate and incubated for further analysis. The proliferation was determined by using Cell Counting Kit‐8 (Dojindo, Kumamoto, Japan), and the absorbance was measured at 450 nm. The results were performed at least three independences.

### Statistical analysis

The difference in AFTPH expression between tumor tissues and normal tissues was compared with an independent t‐test. AFTPH expression in different clinical stages was evaluated using one‐way ANOVA. The relationship between AFTPH expression and patient prognosis was detected using Kaplan–Meier survival analysis and log‐rank test. The correlation between AFTPH and related genes was analyzed using the Pearson correlation test. *P*‐values < 0.05 indicated significance.

## Results

### Expression levels of AFTPH in patients with BRCA, DLBC, LUSC, and PAAD

**Fig. 1:**
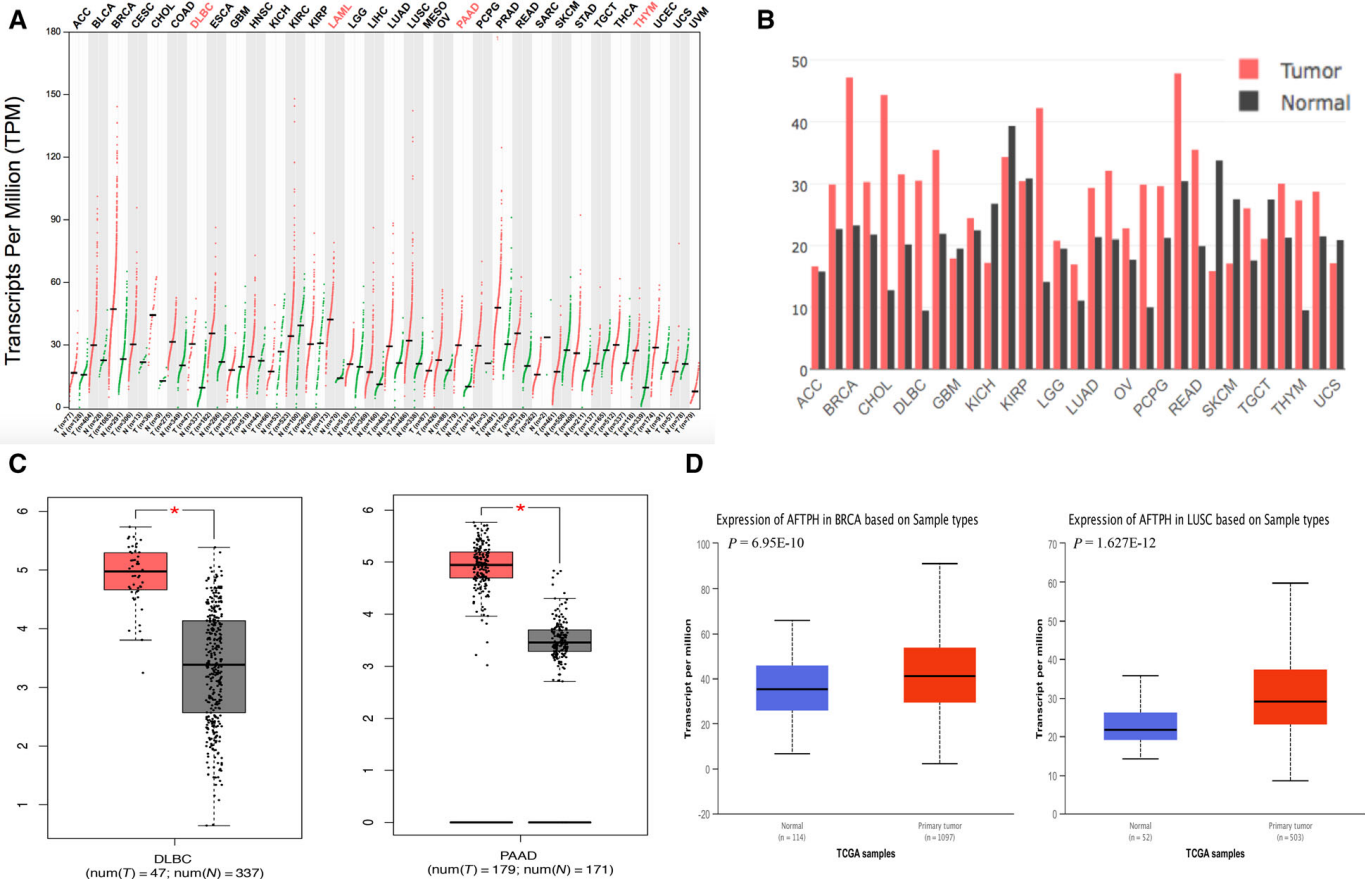
Expression levels of AFTPH in BRCA, DLBC, LUSC, PAAD, and corresponding normal samples. (A–C) AFTPH expression in DLBC, PAAD, and their normal tissues, analyzed by GEPIA. (D) AFTPH expression in BRCA, LUSC, and their normal tissues, analyzed by UALCAN.

**Fig. 2:**
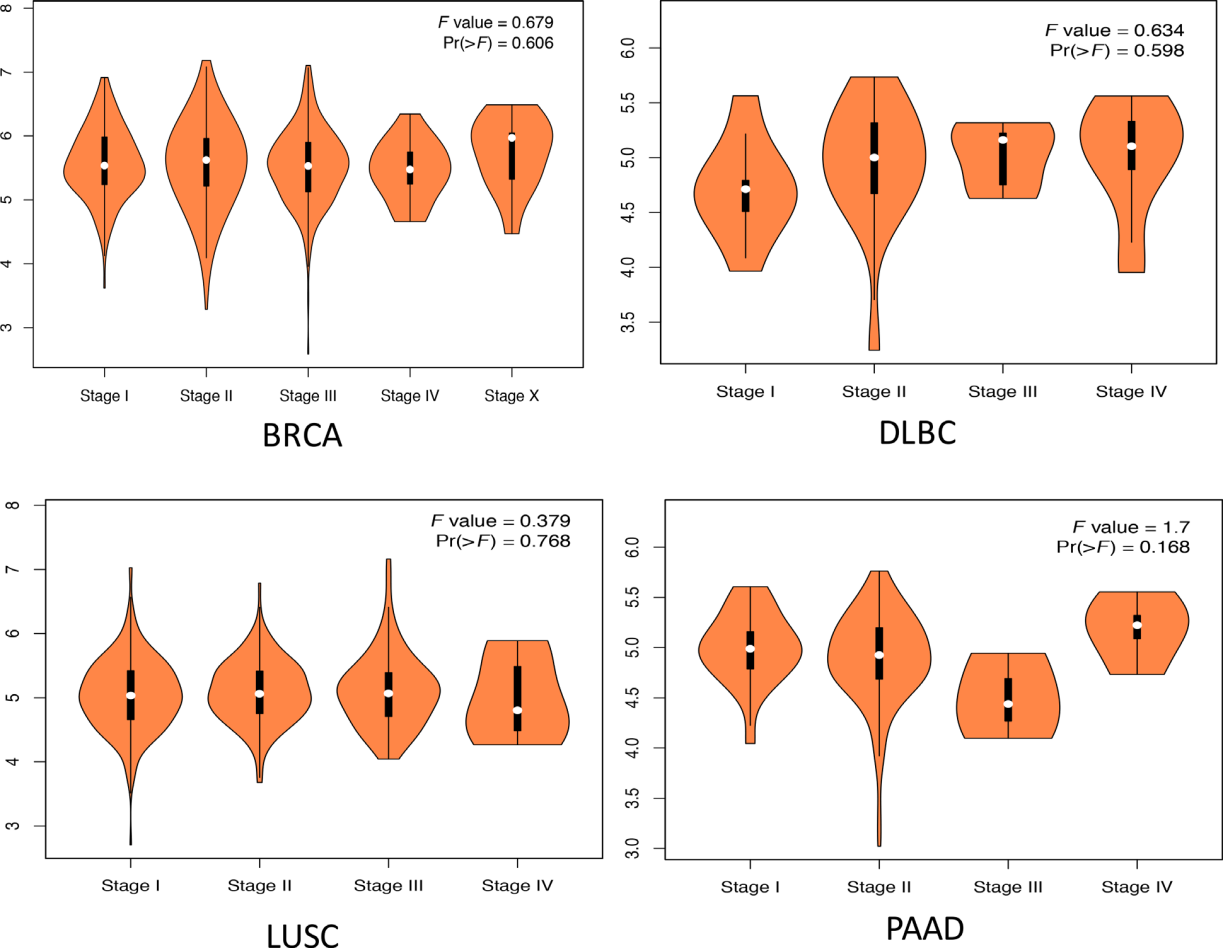
Expression levels of AFTPH in different tumor stages of BRCA, DLBC, LUSC, and PAAD (GEPIA).

**Fig. 3:**
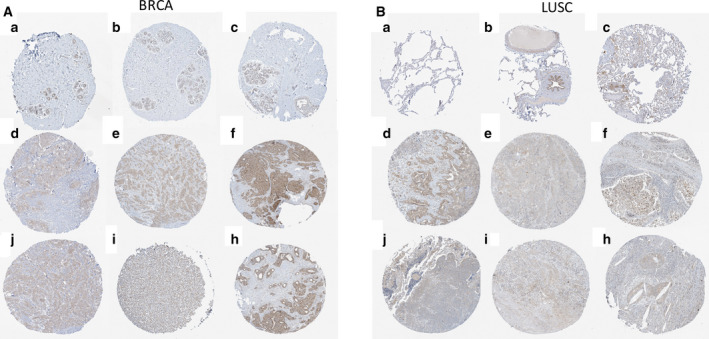
Immunohistochemical staining of AFTPH in BRCA and LUSC (HPA). (A) Immunohistochemical staining of AFTPH in normal breast tissues (a–c) and high expression in BRCA tissues (d–i). (B) Immunohistochemical staining of AFTPH in normal lung tissues (a–c) and high expression in BRCA tissues (d–i).

The expression levels of AFTPH mRNA in tumor samples and corresponding normal samples were analyzed and compared using the GEPIA database. The results showed that AFTPH expression in DLBC and PAAD was higher than that in normal tissues (Fig. 1A–C). In addition, AFTPH expression in BRCA and LUSC was evaluated by the UALCAN database, revealing that AFTPH was overexpressed in the primary tumors (Fig. 1D). The expression levels of AFTPH in different tumor stages were also confirmed through the GEPIA database in BRCA, DLBC, LUSC, and PAAD with no significant difference (Fig. 2). According to the results of immunohistochemistry provided by the HPA database, AFTPH was highly expressed in BRCA and LUSC compared with low expression in adjacent tissues (Fig. 3A,B).

### AFTPH expression in various cancer cell lines

**Fig. 4:**
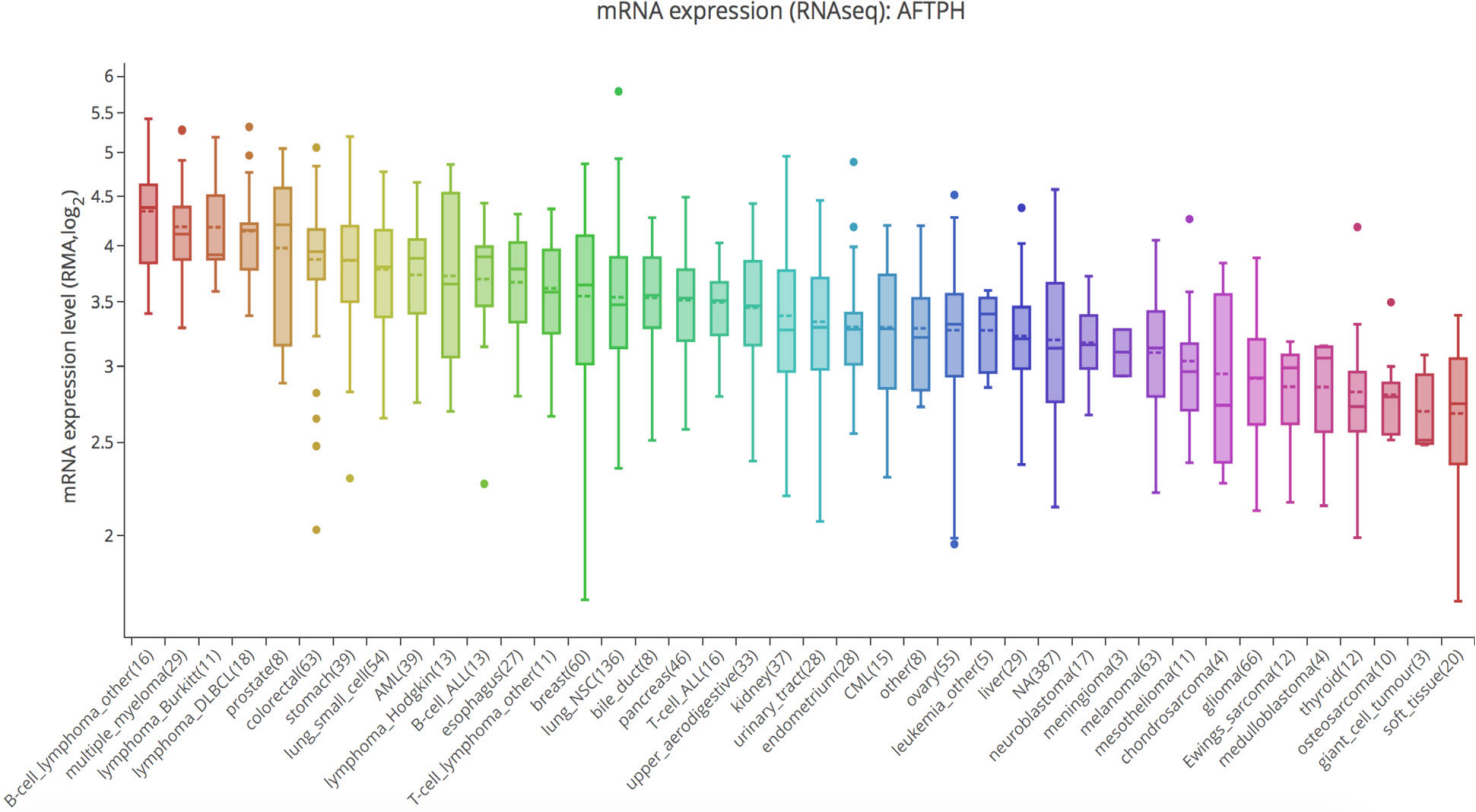
Aftiphilin expression in cell lines of BRCA, DLBC, LUSC, and PAAD analyzed by CCLE.

By collecting genetic information from CCLE, the investigation of AFTPH expression was extended to various cancer cell lines. Similarly, increased expression of AFTPH was found in DLBC, BRCA, LUSC, and PAAD cell lines (Fig. 4). The results verified AFTPH overexpression in lymphoid, lung, abdominal, and breast cancer cell lines.

### Prognostic effect of AFTPH on patients with BRCA, DLBC, LUSC, and PAAD

**Fig. 5:**
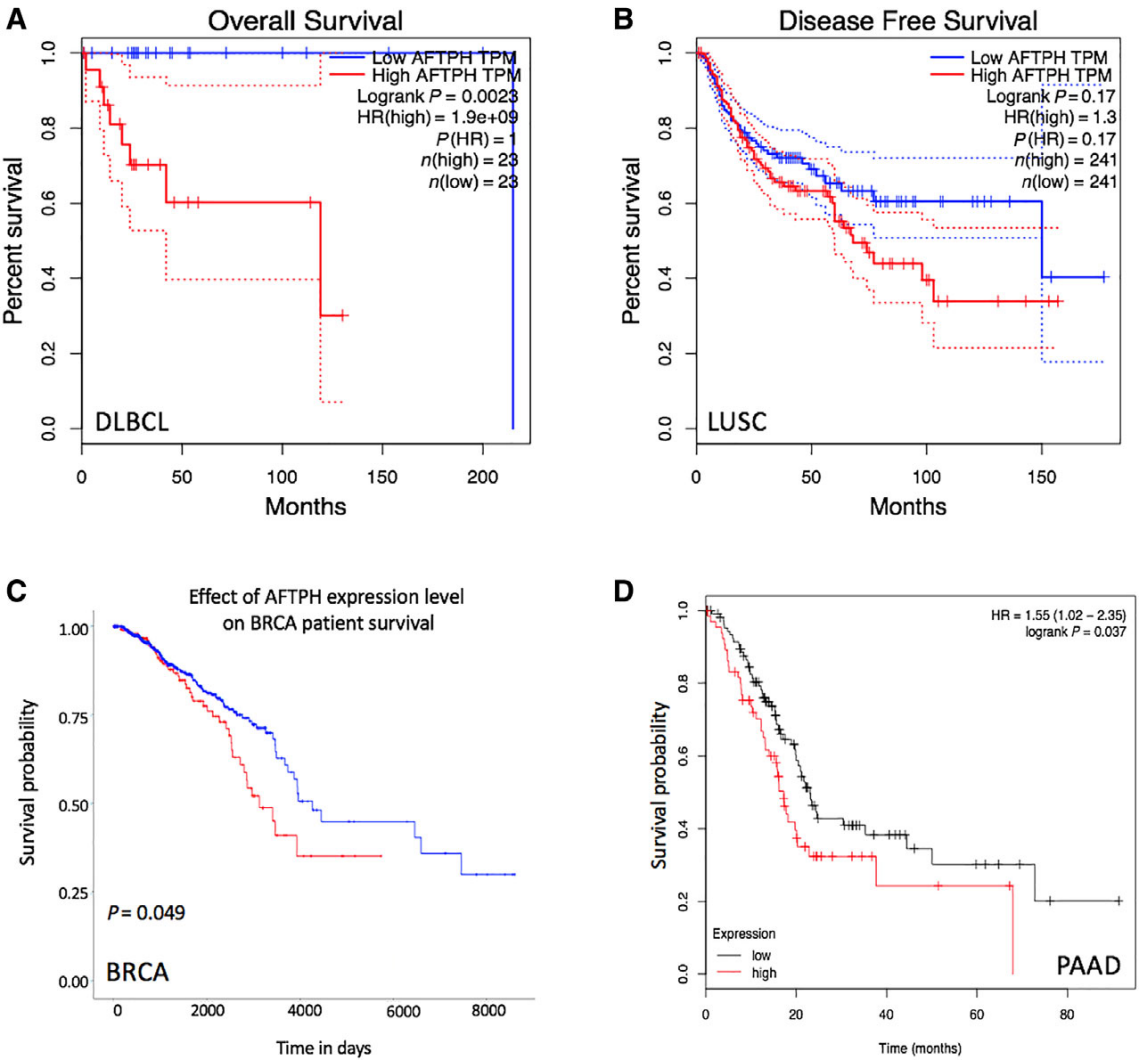
Prognostic significance of AFTPH in BRCA, DLBC, LUSC, and PAAD. (A,B) The prognostic significance of AFTPH in DLBC and LUSC, analyzed by GEPIA. (C) The prognostic significance of AFTPH in BRCA, analyzed by LinkedOmics. (D) The prognostic significance of AFTPH in PAAD, analyzed by Kaplan–Meier plotter.

Whether the expression levels of AFTPH could predict patient prognosis in BRCA, DLBC, LUSC, and PAAD was further studied. The survival analysis formed by the GEPIA database indicated that low expression of AFTPH represented favorable overall survival (OS) in DLBC, as well as better disease‐free survival (DFS) in LUSC (Fig. 5A,B). For BRCA and PAAD, patient information from the UALCAN database (Fig. 5C) and the Kaplan–Meier plotter (Fig. 5D) concluded that lower expression of AFTPH was associated with better survival. The prognostic impact of AFTPH expression on patients with different clinicopathological features in BRCA and PAAD was further explored by Kaplan–Meier plotter in detail (Table 2). High expression was associated with poor OS in female BRCA, white BRCA, stage 2 of BRCA, grade 2 of PAAD, high mutation burden of BRCA, and low mutation burden of PAAD. In contrast, AFTPH overexpression indicated good survival in male PAAD, black BRCA, white PAAD, stage 4 BRCA, grade 1 PAAD, and high mutation burden of PAAD. Overall, AFTPH could be considered to be a reliable prognostic factor.

**Table 2:** Correlation of AFTPH mRNA expression and clinical prognosis in BRCA and PAAD with different clinicopathological characteristics by Kaplan–Meier plotter

Clinicopathological characteristics	Overall survival BRCA (*n* = 1089)			Overall survival PAAD (*n* = 177)		
	*N*	Hazard ratio	*P‐*value	*N*	Hazard ratio	*P‐*value
*Sex*						
Female	1077	1.42 (1.01–2)	**0.042**	80	1.66 (0.93–2.96)	0.086
Male	12	**—**		97	0.54 (0.29–1)	**0.045**
*Race*						
White	752	1.73 (1.18–2.53)	**0.0041**	156	0.61 (0.38–0.99)	**0.044**
Asian	61	0.26 (0.02–2.88)	0.24	11	0.19 (0.01–3.1)	0.19
Black/African American	181	0.41 (0.2–0.85)	**0.014**	2	—	
*Stage*						
1	180	1.92 (0.69–5.32)	0.2	21	2.98 (0.63–14.14)	0.15
2	619	1.68 (1.03–2.76)	**0.036**	146	0.68 (0.42–1.1)	0.11
3	247	0.74 (0.41–1.34)	0.31	3	—	
4	20	0.22 (0.05–0.92)	**0.023**	4	—	
*Grade*						
1	—			31	0.2 (0.05–0.79)	**0.012**
2				94	2.52 (1.4–4.53)	**0.0013**
3				48	0.51 (0.22–1.19)	0.11
*Mutation burden*						
High	493	1.62 (1.01–2.6)	**0.044**	84	0.5 (0.27–0.94)	**0.029**
Low	485	1.34 (0.81–2.2)	0.25	83	2.28 (1.22–4.25)	**0.0079**

Bold values indicate *P* < 0.05.

### Genetic alterations of AFTPH in BRCA, DLBC, LUSC, and PAAD

**Fig. 6:**
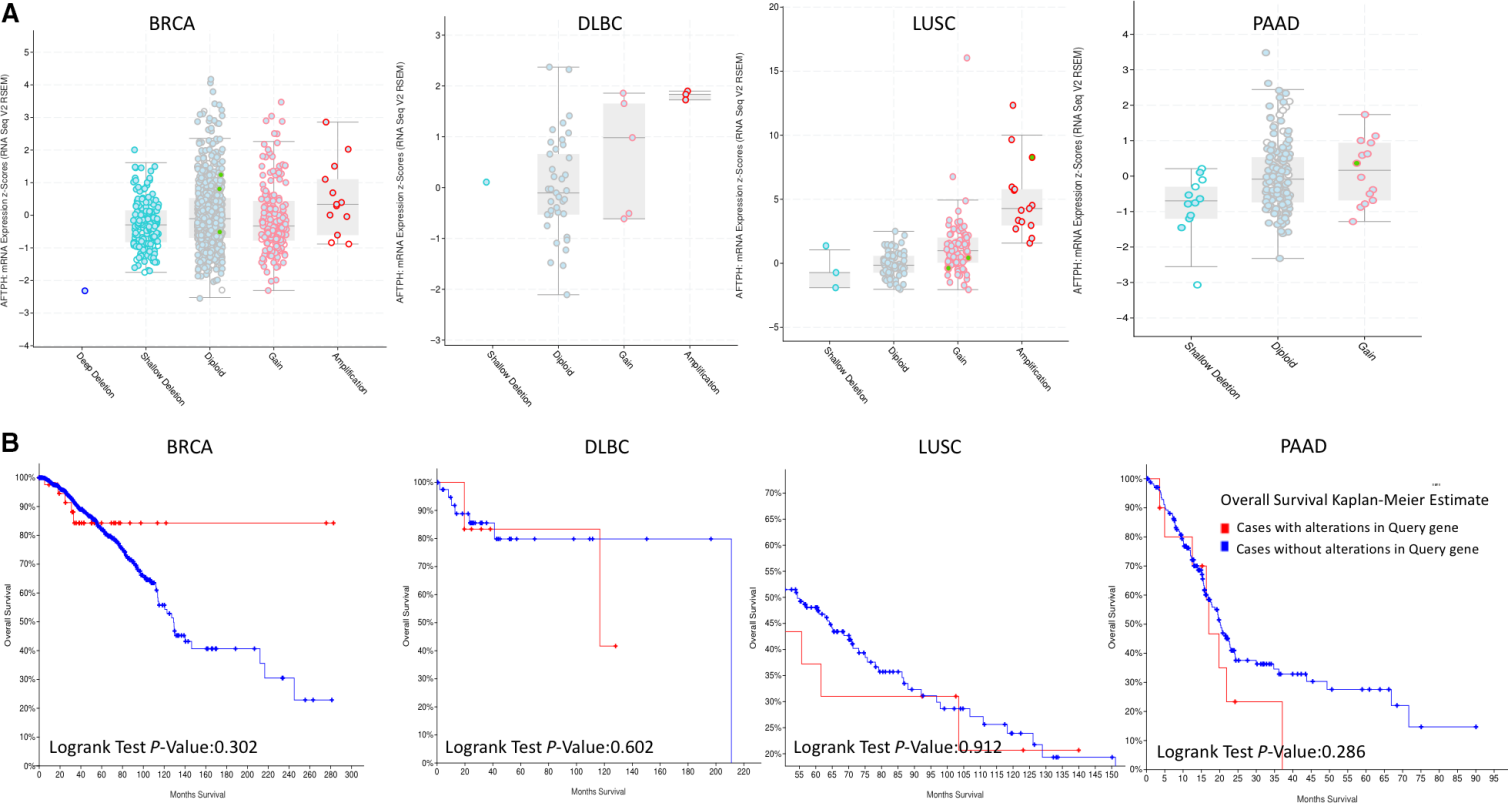
Genetic alterations of AFTPH in BRCA, DLBC, LUSC, PAAD, and patient survival associated with genetic alterations (cBioPortal). (A) Specific genetic alteration types of AFTPH in BRCA, DLBC, LUSC, and PAAD. (B) Relationship between genetic alterations and clinical prognosis in BRCA, DLBC, LUSC, and PAAD.

Genetic alterations of AFTPH in BRCA, DLBC, LUSC, and PAAD were further determined using the cBioPortal website. Figure 6A shows the specific genetic alteration types of AFTPH in BRCA, DLBC, LUSC, and PAAD, which included missense mutation, amplification or gain, deep deletion, and shallow deletion. In particular, Kaplan–Meier survival curves revealed the relationship of alterations and patient OS in BRCA, DLBC, LUSC, and PAAD (Fig. 6B).

### Correlation analysis between AFTPH and other genes and markers

**Fig. 7:**
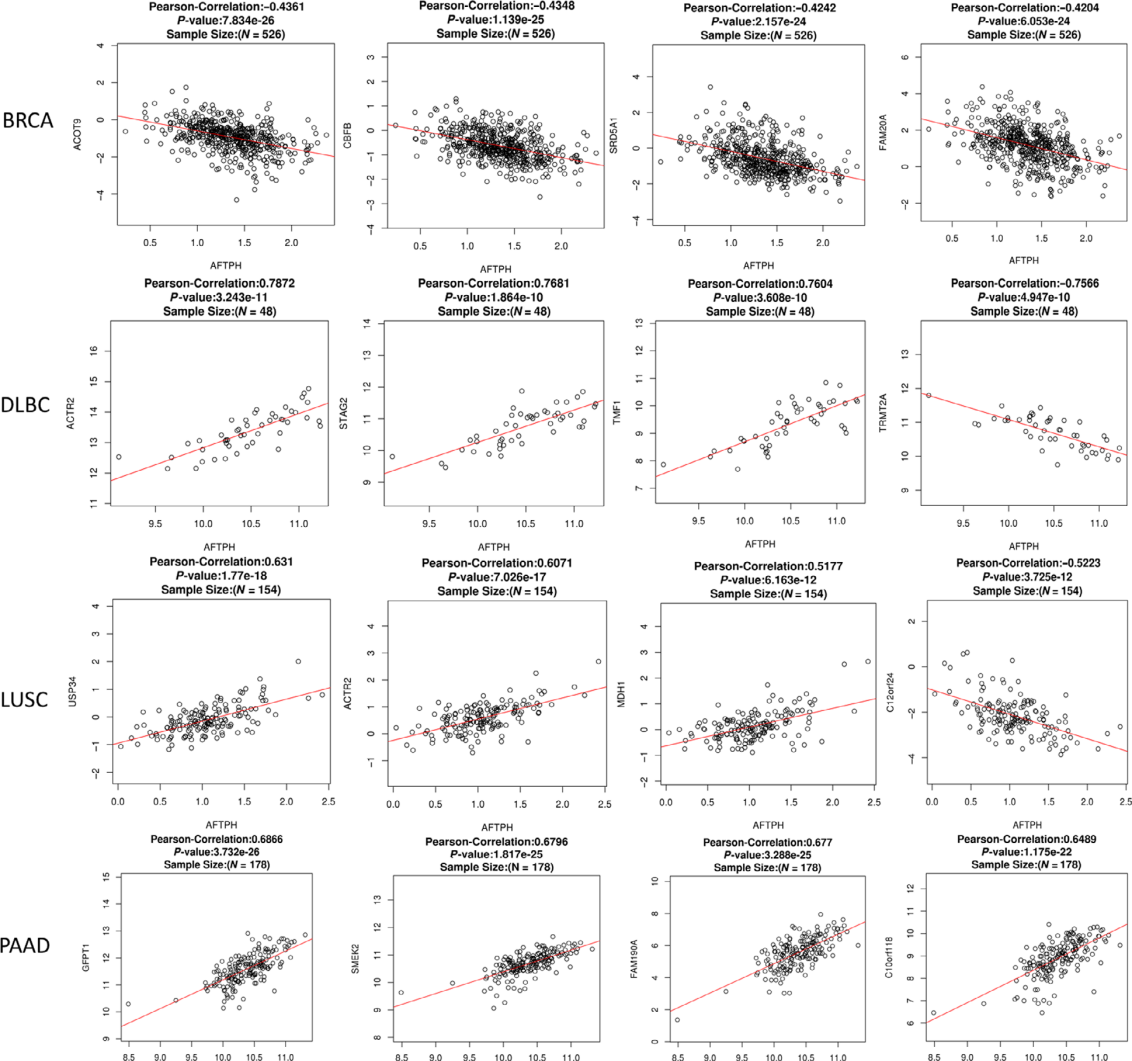
Correlation analysis between AFTPH and various genes and markers in BRCA, DLBC, LUSC, and PAAD (LinkedOmics).

To illustrate the potential mechanisms of AFTPH in BRCA, DLBC, LUSC, and PAAD, correlation analysis between AFTPH and various genes was performed. The scatter plots established by the LinkedOmics database demonstrated that AFTPH expression was negatively related to the expression of ACOT9, CBFB, SRD5A1, and FAM20A in BRCA. In DLBC, AFTPH interacted positively with ACTR2, STAG2, and TMF1 but had a negative interaction with TRMT2A. In LUSC, AFTPH expression was positively associated with USP34, ACTR2, and MDH1 and negatively associated with the expression of C12orf24. In PAAD, AFTPH had a positive correlation with GFPT1, SMEK2, FAM190A, and C10orf118 (Fig. 7). Table 3 also shows that AFTPH expression had a significant interaction with multiple genes and markers in BRCA and DLBC.

**Table 3:** Correlation analysis between AFTPH and related genes and markers in BRCA and DLBC (LinkedOmics)

Gene names	BRCA		DLBC	
	*Pearson*	*P‐*value	*Pearson*	*P‐*value
ST8SIA4	−2.816e‐01	4.808e‐11	4.778e‐01	5.940e‐04
ASS1	−2.185e‐01	4.189e‐07	−5.789e‐01	1.632e‐05
ARFRP1	−2.053e‐01	2.043E‐06	−6.335e‐01	1.345e‐06
CASC4	2.085e‐01	1.416e‐06	4.948e‐01	3.503e‐04
KIAA0232	3.801e‐01	1.601e‐19	4.582e‐01	1.057e‐03
NUTF2	−2.352e‐01	4.782e‐08	−5.685e‐01	2.496e‐05
C14orf79	2.626e‐01	9.625e‐10	−4.531e‐01	1.222e‐03
SRM	−3.804e‐01	1.480e‐19	−4.402e‐01	1.743e‐03
NRBF2	−3.310e‐01	6.431e‐15	4.514e‐01	1.282e‐03
MALT1	−2.086e‐01	1.387e‐06	5.164e‐01	1.717e‐04
RECQL	−3.418e‐01	7.320e‐16	4.439e‐01	1.578e‐03
LEPRE1	−2.810e‐01	5.324e‐11	−5.005e‐01	2.918e‐04
ZNF267	−2.137e‐01	7.540e‐07	6.052e‐01	5.187e‐06
UBE2S	−2.075e‐01	1.591e‐06	−4.590e‐01	1.034e‐03
CAPZA1	−2.283e‐01	1.198e‐07	5.095e‐01	2.168e‐04
POLK	2.037e‐01	2.482e‐06	6.213e‐01	2.454e‐06
NARFL	2.920e‐01	8.463e‐12	−5.726e‐01	2.109e‐05
ACOT9	−4.361e‐01	7.834e‐26	−4.354e‐01	1.979e‐03
PTPRC	−2.737e‐01	1.706e‐10	5.573e‐01	3.888e‐05
PALM	2.333e‐01	6.199e‐08	−5.402e‐01	7.413e‐05
MAPK1	−2.647e‐01	6.931e‐10	6.080e‐01	4.581e‐06
PPP1R14B	‐2.597e‐01	1.482e‐09	−6.485e‐01	6.228e‐07
ITFG3	2.464e‐01	1.032e‐08	−5.705e‐01	2.301e‐05
NDUFA7	2.927e‐01	7.564e‐12	−5.598e‐01	3.521e‐05
ACSL4	‐3.925e‐01	8.011e‐21	4.123e‐01	3.589e‐03
MEGF9	2.326e‐01	6.834e‐08	4.415e‐01	1.680e‐03
PCSK4	2.866e‐01	2.095e‐11	−4.211e‐01	2.877e‐03
ABI1	‐2.315e‐01	7.860e‐08	5.452e‐01	6.148e‐05
CLIP1	2.231e‐01	2.349e‐07	5.557e‐01	4.128e‐05
SDCCAG3	‐2.827e‐01	4.022e‐11	−5.406e‐01	7.288e‐05
MAD2L2	‐2.643e‐01	7.381e‐10	−5.259e‐01	1.238e‐04

### Effect of AFTPH on cell survival in BRCA, LUSC, and PAAD cells

**Fig. 8:**
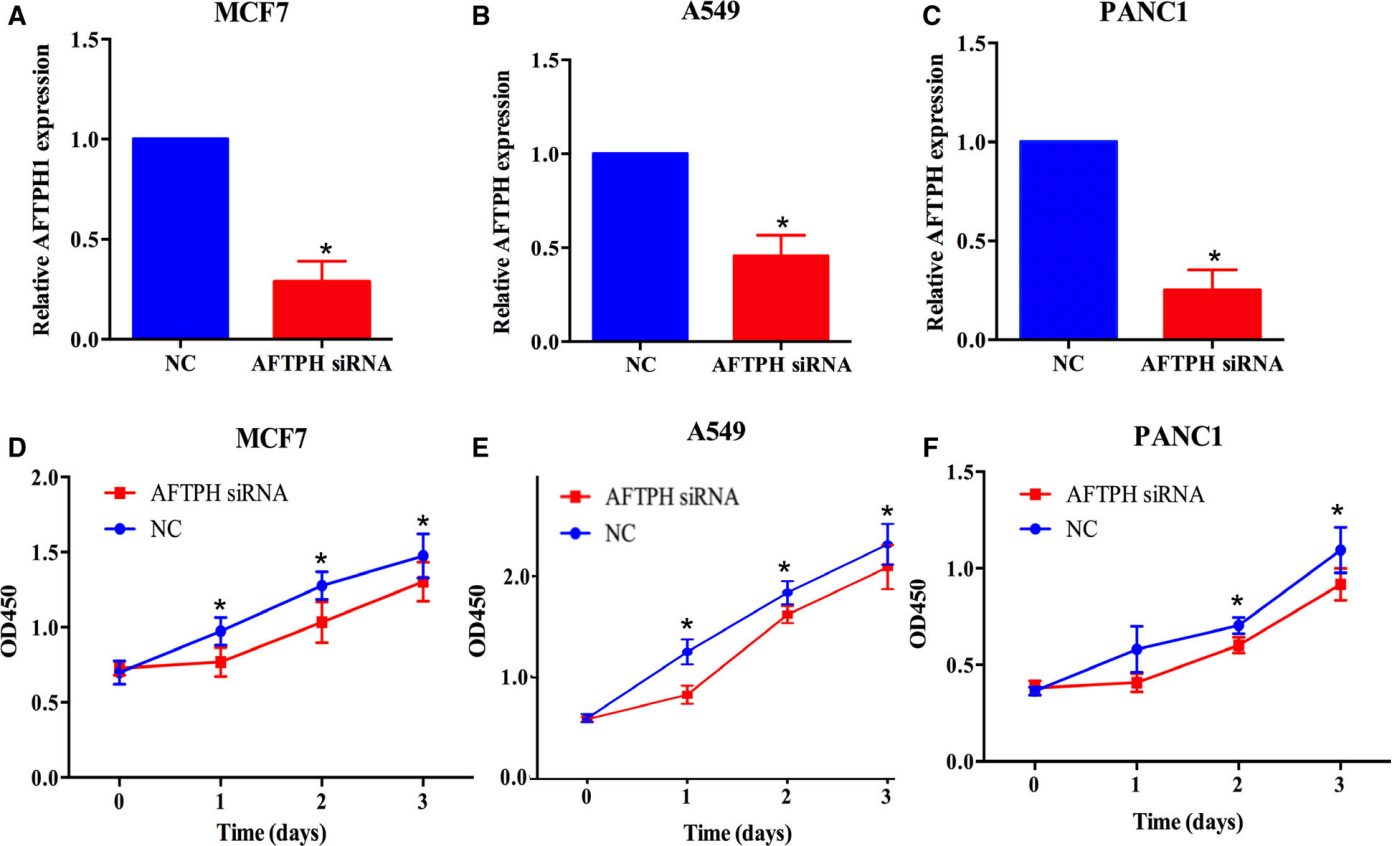
Effects of AFTPH on the proliferation of BRCA, DLBC, and LUSC cells. (A–C) The RT‐PCR results of AFTPH siRNA‐transfected MCF7, A549, and PANC1 cells. Unpaired two tailed *t*‐test was used to accomplish the statistical analysis (*n* = 4). (D–F) The results of the CCK‐8 assay in MCF7, A549, and PANC1 cells transfected with NC siRNA or AFTPH siRNA. And two‐way ANOVA was used to analyze the results (*n* = 3). All graphs are presented as the mean ± standard error of the mean (SEM). *P < 0.05.

Employing a CCK8 assay, we examined whether AFTPH affected the BRCA, LUSC, and PAAD cell lines. The transfected effects of AFTPH siRNA are shown in Fig. 8A–C. The results indicated that downregulation of AFTPH inhibited cell proliferation in MCF7, A549, and PANC1 cells (Fig. 8D–F).

## Discussion

Aftiphilin was initially discovered by Mattera et al., who observed that its core tetrapeptide motif ΨG(P/D/E)(Ψ/L/M) could bind to the γ‐adaptin ear (GAE) domains of AP‐1 and GGA (Golgi‐localized, γ‐ear‐containing, ARF‐binding proteins), which are involved in the pathways of endosomes [2]. Recently, three studies from Law et al. explored the potential mechanisms of the NT/NTR1/miR‐133α/AFTPH axis in colonic epithelial cells and colitis models [7, 8, 9]. Elevated expression of NT and its high‐affinity receptor NTR1 was confirmed in patients with ulcerative colitis [17] and experimental colitis mice [17, 18, 19, 20]. NT/NTR1 signaling could activate ERK, AKT, MARK, and NF‐κB signaling in colonic epithelial cells, inducing inflammation in experimental colitis [21, 22, 23, 24, 25, 26, 27]. Law's studies indicated that activation of NT/NTR1 in colonic epithelial cells and colitis models could induce the expression of miR‐133α and reduce transcription of its downstream target AFTPH, which is involved in inflammatory signals in colonocytes and colitis models [7, 8]. In addition, it was reported that dysregulation of miR‐133α and its target genes could activate ERK [28, 29] and AKT [30, 31] signaling and further promote colorectal cancer development [30, 32, 33, 34]. Therefore, we speculate that AFTPH, the newly discovered miR‐133α target, might play a role in cancer.

In our study, we attempted to characterize the relationship between AFTPH and BRCA, DLBC, LUSC, and PAAD. First, GEPIA database showed the expression differences of AFTPH between tumor samples and normal samples, suggesting that AFTPH was highly expressed in DLBC and PAAD than normal tissues. The UALCAN website was used to assess the expression of AFTPH in BRCA and LUSC. Immunohistochemical images from HPA database also verified positive staining of AFTPH both in BRCA and in LUSC. In addition, the expressions of AFTPH were explored in different tumor stages of BRCA, DLBC, LUSC, and PAAD. Furthermore, AFTPH overexpression in BRCA, DLBC, LUSC, and PAAD was reconfirmed in human cancer cell lines using CCLE.

To further elucidate the possible prognostic value of AFTPH expression in BRCA, DLBC, LUSC, and PAAD, Kaplan–Meier survival curves produced by GEPIA, UALCAN, and Kaplan–Meier plotter were used to compare survival between patients with low AFTPH expression and patients with high AFTPH expression. This analysis indicated that low expression of AFTPH was associated with favorable survival of BRCA, DLBC, LUSC, and PAAD, suggesting AFTPH might be an oncogenetic factor in BRCA, DLBC, LUSC, and PAAD. We also helped to elucidate the effect of AFTPH on OS in 1089 BRCA patients and 177 PAAD patients with different clinicopathological characteristics. Surprisingly, high expression of AFTPH was a protective factor for male PAAD, black BRCA, white PAAD, stage 4 of BRCA, grade 1 of PAAD, and high mutation burden of PAAD. All of these findings demonstrated the potential role of AFTPH in predicting clinical outcomes in BRCA, DLBC, LUSC, and PAAD.

Genetic alterations might be the most important factor affecting cancer development [35, 36]. In this study, cBioPortal database was used as a powerful tool for discovering genetic changes of AFTPH in BRCA, DLBC, LUSC, and PAAD. It should be noted that genetic alterations of AFTPH in BRCA, DLBC, LUSC, and PAAD were not significantly associated with prognostic results. Finally, genes that might interact with AFTPH were discovered by a Pearson correlation test based on the LinkedOmics database. These genes and markers, which were either positively or negatively correlated with AFTPH expression, might be involved in the development of AFTPH‐related cancers. Furthermore, we conducted experiments by transfecting BRCA, DLBC, and LUSC cell lines with AFTPH siRNA. The results showed that AFTPH promoted proliferation in BRCA, DLBC, and LUSC cells, which is consistent with our previous informatics analysis.

## Conclusions

To the best of our knowledge, this study was the first to comprehensively analyze the expression profiles and prognostic role of AFTPH in solid tumors and hematological malignancies, as well as providing evidence for subsequent studies on the molecular mechanism governing this role. AFTPH was suggested to be a potential therapeutic target and prognostic biomarker for BRCA, DLBC, LUSC, and PAAD. However, there are still several limitations to these results, and the molecular mechanisms governing the AFTPH‐related cancer pathway should be further elucidated.

## Conflict of interest

The authors declare no conflict of interest.

## Author contribution

TZ and YC contributed to this article in the aspects of drafting the work and analyzing the data for the work. ZL was revising the paper. YL and YT made substantial contributions to the conception and design of the work.

## Data Availability

The data provided in the manuscript will be available from the corresponding author upon reasonable request.
